# Portuguese nurses’ perceptions of how educational models influence professional identity and practice

**DOI:** 10.1590/0034-7167-2025-0262

**Published:** 2026-09-18

**Authors:** Sandra Rita Pereira Fernandes, Márcia Maria Carneiro Oliveira, Maria de Fátima Carneiro Pereira, Maria João Cardoso Teixeira, Olga Maria Pimenta Lopes Ribeiro, Tânia Marisa Pinto Rodrigues, Cânia Patrícia Torres, Beatriz Rodrigues Araújo

**Affiliations:** I Universidade do Porto. Porto, Portugal; II Universidade Federal da Bahia. Salvador, Bahia, Brazil; III Royal National Orthopaedics Hospital NHS. London, United Kingdom; IV Universidade Católica Portuguesa. Porto, Portugal

**Keywords:** Leadership, Nursing, Faculty, Teaching, Professional Identity., Liderazgo, Enfermería, Docentes, Enseñanza, Identidad Profesional.

## Abstract

**Objectives:**

to understand how the Traditional and Social Constructivist models used by nurse educators influence Portuguese nurses’ professional identity formation and clinical practice.

**Methods:**

descriptive, qualitative study guided by the ego-ecological theory of Zavalloni and Louis-Guérin. Data were generated by exploring responses to the Psychosocial Identity Inventory in representational units of identification with and differentiation from nurse educators, complemented by interviews conducted using recommended techniques.

**Results:**

nurses identified with educators aligned with the Social Constructivist Model and distanced themselves from or opposed those aligned with the Traditional Model. Findings revealed leadership roles and obstacles within the identification and differentiation groups.

**Final Considerations:**

within the Social Constructivist Model, the influence of educators on nurses’ professional identity formation and clinical practice proved more effective. Encouraging nurses to assume a critical and transformative role supports professional development and strengthens a culture of leadership oriented toward the quality of care and innovation.

## RESUMEN

**Objetivos:**

comprender cómo los modelos de formación docente tradicional y socioconstructivista influyen en la construcción de la identidad profesional y la práctica clínica de enfermeros portugueses.

**Métodos:**

estudio descriptivo, cualitativo, guiado por la teoría ego-ecológica de Zavalloni y Louis-Guérin. Los datos se obtuvieron de respuestas al Inventario de Identidad Psicosocial, en unidades representacionales de identificación y diferenciación frente a docentes de Enfermería, y se complementaron con entrevistas según técnicas recomendadas.

**Resultados:**

los enfermeros se identificaron con docentes del Modelo Socioconstructivista y se diferenciaron de u opusieron a los del Modelo Tradicional. Los hallazgos permitieron reconocer formas de liderazgo ejercidas y obstáculos en los grupos de diferenciación e identificación.

**Consideraciones Finales:**

en el Modelo Socioconstructivista, la influencia docente sobre la identidad y práctica clínica resultó más efectiva. Promover un papel crítico y transformador favorece desarrollo profesional y fortalece una cultura de liderazgo orientada a calidad del cuidado e innovación.

## INTRODUCTION

In today’s healthcare context, where growing complexity and the need for innovation and development shape the delivery of high-quality nursing care, nurse educators play a key role in helping students develop critical thinking, pursue knowledge, and integrate theory and practice. Achieving this integration in nursing education remains an ongoing challenge, one that can be addressed through collaborative efforts among nurse educators, clinical nurses, and students^(1)^. Nurse educators can also exercise leadership in their relationships with students and clinical nurses, influencing the delivery of person-centered, evidence-based care and strengthening partnerships with nurse researchers in clinical practice^(2)^. To exercise leadership effectively, these educators must remain aware of their role models and points of reference. They demonstrate this through ethical conduct, an assertive approach to teaching professional responsibilities, and the way they interact with patients, nurses, and other members of the multiprofessional team^(2)^.

Against the backdrop of ongoing changes in Portugal’s healthcare system, it is essential to understand the extent to which the educational models adopted by nurse educators, whether grounded in the Traditional Model or the Social Constructivist Model, may reinforce institutional alignment with the marketization of healthcare or promote professional autonomy and humanized care^(3)^.

The traditional teaching and learning model is teacher-centered, with the educator positioned as the authority who transmits knowledge, leaving the student in a largely passive role. In the Social Constructivist Model, students, educators, and the social environment all play a role in knowledge development^(4)^ and in nursing education. This model emphasizes the value of students’ expectations, prior knowledge, and experiences through proactive learning methods, while positioning the educator as a facilitator within this approach^(5)^.

Collaboration between nurse educators and clinical nurses in clinical education settings fosters an environment for sharing knowledge and experiences and encourages change. These “practice-based learning communities” offer several advantages, including contextualized learning, engagement, collaboration, trust, and integration among nurse educators, students, and clinical nurses^(6-8)^.

In nursing education, partnerships among nurse educators, nurse supervisors, and tutors connect complementary forms of knowledge that are fundamental to educating future nurses^(8)^. Clinical education plays an important role in the socialization and professional identity formation of nursing students, a process in which clinical nurses play a central role and also serve as professional role models for future nurses within a collaborative learning environment^(7)^. By exercising and teaching leadership-that is, by influencing nurses and future nurses-nurse educators aligned with the Social Constructivist Model help these professionals recognize and assume their leadership potential, promoting innovation, change, and improvements in care delivery, healthcare services, and professionalism^(9)^.

Although studies have addressed the need to transform nursing education through the Social Constructivist Model^(6,10-12)^, no studies were found that examine, from nurses’ perspectives, how the educational models used by nurse educators influence professional identity formation and nursing practice.

The ego-ecological theory of Zavalloni and Louis-Guerin^(13)^ provides a way to understand the construction and reconstruction of professional identity as a dynamic, situated process that takes into account individuals’ interactions with the various social and institutional contexts in which they are embedded. This perspective encompasses cognitive processes such as information selection and retention, as well as phenomena related to social influence and attribution. In this way, it links past, present, and future through processes of recoding, evaluation, and orientation within nurses’ professional environment, where both achievements and setbacks are experienced. Subconscious contents remain at the periphery of awareness, yet they influence identity trajectories and professional practice. This framework is particularly well suited to analyzing how the different educational models used by nurse educators shape Portuguese nurses’ professional identity formation and practice by bringing together the individual, relational, and ecological dimensions.

In light of the above, the following research question was posed: “How do educational models used by nurse educators influence Portuguese nurses’ professional identity formation and clinical practice?”

## Study relevance

This article is based on a doctoral dissertation^(14)^ available in the institutional repository (http://hdl.handle.net/10400.14/36698).

## OBJECTIVES

To understand how the Traditional and Social Constructivist educational models used by nurse educators influence Portuguese nurses’ professional identity formation and clinical practice.

## METHODS

### Ethical considerations

This study complied with the ethical principles for medical research involving human subjects set forth in the Declaration of Helsinki, was approved by the Ethics Committee of the *Universidade Católica Portuguesa*, and followed all applicable ethical procedures. All participants signed the Informed Consent Form (ICF) after reading the study information and having any questions clarified. Participation was voluntary and occurred only after explicit agreement and signed informed consent.

### Theoretical and methodological framework

The study was grounded in ego-ecological theory, which views individuals as embedded in a social environment shaped by belonging and interactions with social groups, where internal emotional memory is influenced by processes of identification (Self) or differentiation (Alter) in relation to others in that environment. From this perspective, emphasis is placed on the impact of identification with others in one’s immediate surroundings^(13)^. By adopting this framework, the study explored how nurse educators contribute to nurses’ professional identity formation by influencing their emotional experiences, beliefs, and self-perceptions. It also made it possible to understand the emotional dynamics of the academic learning environment and how they shape identity development.

### Study design

This was a qualitative study grounded in the ego-ecological theory of Zavalloni and Louis Guérin^(13)^. The study followed Enhancing the QUAlity and Transparency Of health Research (EQUATOR) Network guidelines and used the Consolidated Criteria for Reporting Qualitative Research (COREQ) checklist.

### Sample

Purposive sampling combined with the snowball technique^(15)^ was used to obtain in-depth data. The sample included 19 nurse tutors and/or supervisors who met the following inclusion criteria: having 10 or more years of professional experience and working in specialized care or primary care settings in Portugal. The snowball process began with two initial contacts, who referred other professionals from their networks, progressively expanding the sample to include nurses from different institutions and with diverse experiences. The interviewer, who conducted all interviews, had experience in qualitative research since 2004 and formal training in ego-ecological methodology since 2014. She was also a nurse educator with a PhD in Nursing and had a professional relationship with the first two interviewees, who were nurse colleagues at the institution where she had previously worked as a nurse.

### Data collection instrument

Participants were initially invited via email and provided with information about the study. Once consent had been obtained, meeting dates and locations were scheduled according to participants’ preferences. Each meeting involved only the principal investigator/interviewer and the interviewee. Locations varied and included participants’ homes, cars, and rooms in healthcare facilities.

On the day of data collection, participants were asked to complete the adapted Psychosocial Identity Inventory (*Inventário de Identidade Psicossocial*, IIP)^(16)^, which served as a prompt for their responses and was then explored in an interview lasting 60 to 240 minutes. Through the IIP, each interviewee was asked to identify with or differentiate themselves from the groups “We...” and “They...”. In this study, “We” referred to nurses, whereas “They” referred to the group of nurse educators with whom participants had interacted or continued to interact. This process generated representational units, or first-degree data, that expressed identification or differentiation between the Self (nurse) and the Alter (nurse educator).

These associations are intended to elicit sequences linking personal and collective content, which are then used to evoke additional content^(13)^. Subsequently, following the technique recommended by the inventory’s authors, the first-degree data were used as prompts during the interview, generating secondand third-degree data through focused introspection. This process made it possible to identify social relationships with the Other/Alter-represented for these nurses by the nurse educator-and their emotional influence on the construction of the professional nursing Self.

Within the subject’s Operative Inner Environment, long-term memory is stored and can emerge under conditions of minimal stimulation. Access to it occurs through the meanings attributed to these representational objects of the Self and the Other. This interpretive process unfolds through the different meanings of belonging and differentiation, their positive, negative, or neutral valence, the subgroups involved, their application to the Self, and the importance assigned to these representations, using the Representational Contextualization Method (*Método de Contextualização Representacional*, MCR)^(13)^. Accordingly, the interview was conducted using the MCR and Associative Network Analysis. The material produced by participants was used to clarify the meaning of the data and their categorizations and representations, making it possible to align personal and collective history and experience through an understanding of how personal lived experience connects with social lived experience. In this way, representational units emerge into consciousness-images, people, situations, and memories that usually remain outside the subject’s awareness but are brought forth by the prompts^(13)^. The procedure was repeated until data saturation was reached.

### Data analysis

Data were analyzed using ego-ecological methodology. The analytic process involved categorizing the interviews through comparative analysis, interpreted in light of the Traditional Model and the Social Constructivist Model^(4)^. This approach was intended to elucidate the underlying dynamics and predominant themes related to the educational models perceived by participants. Interviews were audio-recorded, labeled ID1 to ID19, transcribed verbatim, and returned to participants for comment or revision within two weeks. If no comments were received, participant agreement with the content was assumed. Thematic analysis was conducted by three researchers using Braun and Clarke’s methodology^(15)^, with support from NVivo. Categories, subcategories, and codes were developed systematically until data saturation was reached^(12)^. To ensure rigor and analytic integrity, three authors, all specialists in thematic analysis with more than 10 years of experience and involved in several nursing education research projects, collectively reviewed the categories and subcategories derived from coding and analysis, maintaining a reflexive approach throughout the process. The final framework comprised two categories: Perceptions of the Nurse Educator Profession and Advantages and Disadvantages. The latter was subdivided into Relational Competencies, Teaching Competencies, and Scholarly Competencies. The full set of categories, subcategories, and corresponding codes was discussed and validated by the rest of the research team.

## RESULTS

Data analysis showed that a larger proportion of meaning units fell under the Traditional Model than under the Social Constructivist Model.

Nineteen experienced nurse tutors and/or supervisors with different academic backgrounds and exposure to different teaching models were invited, and all agreed to participate, resulting in a final sample of 19 participants. Thirteen had graduate education, whereas six held only a bachelor’s degree in Nursing. The nurses had a mean of 17.8 years of professional experience and a mean age of 40 years; 13 worked in hospitals and 6 in health centers.

Use of the IIP generated first-degree data reflecting processes of positive, negative, and neutral identification with and differentiation from the nurse educator group. Analysis of the interviews further explored these evocations and revealed meanings linked to relationships with nurse educators, particularly the educational model they embodied. Identification with educators aligned with the Social Constructivist Model and differentiation from those aligned with the Traditional Model were observed. Ego-ecological theory^(13)^ helped clarify how interactions with nurse educators and their teaching models, as significant Others, support nurses’ identity transitions.

**Figure 1:**
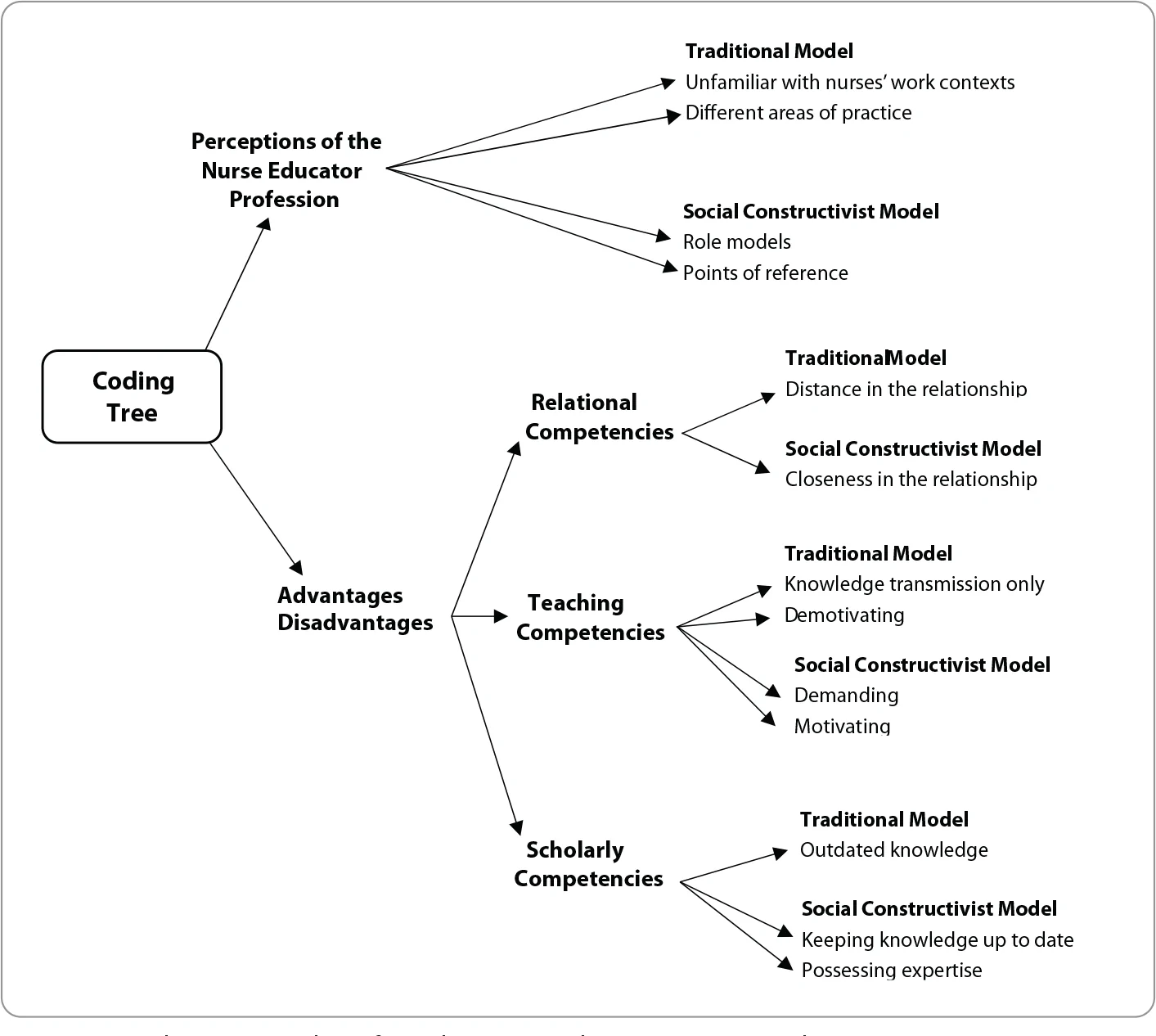
Coding Tree resulting from thematic analysis, Porto, Portugal

Based on this analysis, a diagram (Figure 1) was developed to provide a clearer representation of the positions and content reported by participants.

Based on responses to the Psychosocial Identity Inventory, it became clear that nurses positioned themselves along a continuum between differentiation from and identification with nurse educators, predominantly revealing identity-related ambiguities toward this professional group, associated with the educational models these educators adopted: the Traditional Model or the Social Constructivist Model.

### Traditional Model

Data analysis showed that differentiation predominated among the interviewed nurses in how they perceived the Nurse Educator Profession and in the Relational Competencies, Teaching Competencies, and Scholarly Competencies associated with the Traditional Model, as shown in Figure 1.

### Perceptions of the Nurse Educator Profession

Differentiation was most evident within the category Perceptions of the Nurse Educator Profession, in which nurse educators were perceived as unfamiliar with nurses’ work contexts and as working in areas different from those in which nurses practice.

The interviewed nurses differentiated themselves from nurse educators mainly because they viewed them as distant from and unfamiliar with their work contexts:

[…] *generally speaking, and of course, there are exceptions; nurse educators have, in general, ceased to be nurses as far as care delivery is concerned.* (ID6)

[…] *it is telling that when a nurse goes on to devote themselves exclusively to the school, they move from the Ministry of Health to the Ministry of Education, which says a great deal about what it means to be a nurse educator: someone who is in a school teaching nursing but who ultimately develops a disconnect from direct patient care.* (ID10)

The theory-practice divide remains unresolved, revealing identities grounded in theory disconnected from practice, attributed to nurse educators, and identities grounded in practice informed knowledge, as asserted by nurses:

[…] *because a theorist is someone who knows a body of knowledge and then cannot put it into practice, cannot be versatile, cannot apply it to the person in front of them across different contexts. Theory is important across a range of scientific fields, and of course, it is important in what I do; but a theorist is someone who cannot move beyond theory and make it meaningful in practice.* (ID11)

*We need more nurse educators who are nurses and fewer theoretical nursing educators who went straight into theory, and that creates a certain mismatch, and they fall a little short!* (ID8)

In these nurses’ view, there was no consensus as to whether distance from direct patient care is an advantage or a disadvantage for nursing education today. Even so, most meaning units pointed to the difficulties this distance creates for teaching and for students:

*I think theory and practice should go together, but at this point, since nurse educators are only in schools teaching, I see them working better as separate things.* (ID13)

*Nowadays, some schools are already changing their methods and bringing the school educator and the clinical preceptor into closer coordination. There is greater closeness to the clinical preceptor and to clinical practice; most clinical placements are supervised by the school educator together with a practice supervisor or tutor.* (ID10)

The nurses even recognized that they themselves were the product of the split between theoretical nursing and practical nursing, because they felt that nurse educators called for the mere application of theory to practice rather than the integration of these components of knowledge, which created difficulties for them, as professionals, in delivering care:

[…] *it gives you the sense that, when you are with nurse educators at school, everything is very linear, that you are going to follow all those steps exactly, that line of reasoning, that line of thought that “They” are working with; but then, in reality, once you get there, it depends a great deal on the person in front of you, your working conditions, and the leadership of the unit managers.* (ID14)

*There is a huge separation between the theory left behind at school and practice, which they then stop understanding; and I think “We” end up being the product of all this, because we are then evaluated by someone from the academic side who does not know us, and there has been a separation.* (ID18)

Within the category Perceptions of the Nurse Educator Profession, some interviewees considered nursing and nurse education to be distinct professions, especially because they involve different areas of practice:

[…] *they are two different groups because they work in different areas and with different purposes. One works with the patient and family as the focus of care; the other has the student as the focus of their work-the client is different!* (ID1)

*I do not regard them as fully healthcare professionals because they are outside the care delivery context. “They” teach care delivery, but they do not practice it. “We” are nurses who provide care in our own practice, and “They” are nurses who do not provide that care, but teach it.* (ID5)

### Deficits in Relational Competencies

Competencies also emerged as a point of differentiation between nurses and nurse educators, with deficits in Relational Competencies predominating, followed by deficits in Teaching Competencies and Scholarly Competencies within the Traditional Model.

Differentiation between nurses and nurse educators was evident in the dynamics of interpersonal relationships and in assessment methods.

Nurses described the educator-student relationship as distant, hierarchical, and rigid:

*In terms of attitude, in the way they related to us, in that lack of closeness, in what they expected from “Us,” there was no flexibility toward the student-it was that way, and it had to be that way!* (ID2)

[…] *the people there end up thinking they belong to a different elite from everyone else, but that does not mean they are actually any better than the rest. There is a bit of that school versus-school divide, those conflicts that sometimes get passed on to professionals.* (ID9)

The threatening nature of assessment was identified as one of the causes of this relational distance:

[…] *being put to the test, being pressured-it felt as though they were humiliating us* […]. (ID3)

[…] *as a student, I had certain fears because we were being assessed.* (ID4)

### Deficits in Teaching Competencies

Deficits in Teaching Competencies were also identified in nurse educators, who were perceived as transmitters of knowledge and as demotivating. Nurses took a dim view of teaching based solely on transmissive methods:

[…] *not very dynamic; they are the ones who go in there just to rattle things off, dump the content, and that is it!* (ID19)

[…] *I had some and thought: I did not learn from this one! With this one, it was just content dumping!* (ID7)

Participants in this study distanced themselves from nurse educators whose criticism and way of teaching demotivated students:

*The criticisms were not motivating; they were not meant to build something better. They were criticisms that did little to motivate and, at times, made you want to give up.* (ID4)

[…] *they are those nurse educators who say monotonous things, spark no interest, and just dump information in a monotone way.* (ID8)

### Deficits in Scholarly Competencies

Distance from and opposition to nurse educators were observed when they failed to keep their knowledge up to date across different areas of nursing or in their teaching planning:

[…] *it was as if their understanding of nursing knowledge had become stuck in the past, which is completely at odds with what “We” know is happening in healthcare.* (ID6)

[…] *I realized there were nurse educators who taught one course one year and a different one the next, and the notes were not their own; they just kept repeating the same materials for years without updating them.* (ID18)

### Social Constructivist Model

Data analysis showed that nurses identified with nurse educators mainly in terms of their Perceptions of the Nurse Educator Profession and their Teaching Competencies, followed by Relational Competencies and Scholarly Competencies (Figure 1), in keeping with the Social Constructivist Model.

### Perceptions of the Nurse Educator Profession

Two related yet distinct lines of identification emerged. On the one hand, nurses who viewed nurse educators as models tended to idealize them and showed little critical reflection in doing so. On the other hand, nurses who took nurse educators as points of reference did so more critically and with some degree of distance.

The accounts in which nurse educators were described as models expressed positive differentiation, understood as an influence regarded as important in students’ education and in their lives:

[…] *when I do not know something, there is a group of people I can turn to and learn from who, in principle, hold certain knowledge that can meet my conceptual needs; “They,” in principle, will have that knowledge, will already have studied, will already have developed expertise, or will hold knowledge that I do not yet have!!* (ID17)

[…] *being a nurse educator requires a level of knowledge and teaching beyond basic training, so I see them as people with somewhat greater intellectual ability, able to acquire knowledge and then adapt and convey it to students so that they can learn.* (ID12)

Nurses who viewed nurse educators as points of reference understood this characteristic more critically, as something that supported their development, especially during the transition to professional practice:

[…] *educator X really was my model as a nurse, until I realized that he could no longer be! Because he was someone so closely tied to teaching, and for so long, that I could no longer recognize in him my own idea of what a nurse should be, although I still consider him a person of outstanding competence.* (ID6)

[…] *to guide us in the pursuit of knowledge, like guides helping us find our way.* (ID15)

Nurses reported identifying with nurse educators because they saw the two roles as complementary: “They” possessed “theoretical” knowledge, whereas nurses possessed “practical” knowledge. Thus, the two professions complemented one another, revealing not so much identification with these professionals as positive differentiation:

*Educators are the ones with the knowledge; when I speak of “Masters,” I mean theoretical knowledge, whereas nurses are more hands-on. Educators may have a great deal of theory, and many of “Us” also have a great deal of theory while living much more in practice, so the two complement each other.* (ID7)

[…] *because, deep down, nurse educators reflect what we are. They are the ones training nurses and helping us become qualified professionals.* (ID3)

### Teaching Competencies

Within Teaching Competencies, interviewees identified with nurse educators they perceived as motivating and demanding:

[…] *they spark curiosity about the topic; they motivate you, and you go looking for something on it, or they make you go look for it!* (ID19)

[…] *they create greater motivation in their students-the kind who bring out students’ potential, motivate them, and help them grow and develop their knowledge. If “They” are stimulating, then they foster motivation.* (ID8)

Although, as students, they had regarded these demands as a source of differentiation, they now recognized, as nurses, that those same demands had fostered a desire to improve and do better, making them a point of identification with nurse educators.

### Relational Competencies

In Relational Competencies, identification with nurse educators was reflected in the closeness established in the relationship. Participating nurses valued a close relationship with nurse educators, marked by a developmental journey and by the strengthening of students’ potential:

*It is about getting to know the student, having a certain closeness-not intimacy, but enough closeness to know the student, to know their weaknesses and strengths, so that you can then help them grow; this applies both to clinical education and to teaching, or else to motivating “Us,” as students, along that developmental path.* (ID1)

[…] *it is about respecting the other person’s space, respecting the other person’s opinion. And respect is also fundamental in the educator-student relationship.* (ID5)

### Scholarly Competencies

Within Scholarly Competencies, identification centered first on Perceptions of the Nurse Educator Profession-mainly on seeing nurse educators as possessing knowledge-and later on knowledge updating. What had initially been classified as identification within Scholarly Competencies later came to be interpreted as positive differentiation. Nurses described nurse educators as possessing knowledge that they themselves did not have:

*At present, they are people who possess a different kind of knowledge that “We” do not have. They have studied more; in terms of knowledge, they have another kind of competence that “We” do not have.* (ID16)

*It is a person who holds scientific knowledge and is going to give “Us” more knowledge, right? More skills!* (ID1)

Nurses also recognized the constant updating of knowledge as a competency of nurse educators with which they identified:

*They have to be constantly engaged in research, constantly producing knowledge, constantly updating themselves; they have to be very proactive in their work.* (ID9)

*They are always updating themselves; they study the latest evidence in order to teach.* (ID3)

## DISCUSSION

This study is grounded in Social Constructivism. It draws on Social Representation Theory and, more specifically, on Zavalloni and Louis-Guérin’s Psychosocial Identity Theory, in which social representations are understood as dynamic and constantly reshaped, influencing behavior^(13)^. Accordingly, the interaction between “We” and “They” centers on knowledge of groups of belonging and non-belonging-that is, identifications and oppositions/differentiations, as well as the meanings constructed by the subject. In addition, recognition of the significant Other is important for understanding identity, practices, and professional development.

Zavalloni and Louis-Guérin’s psychosocial perspective^(13)^ implies a contextual, integrative approach that encompasses the various phenomena shaping identity reconfigurations, from education to work, while incorporating changes in social, political, and economic contexts and, more specifically in this study, the prevailing educational models.

Education, work, and identity are intertwined, shaping socialization and preparation for the nursing profession during training, which in turn enables the transition to professional practice. However, the influence of education does not end there; it extends throughout professional development, in both formal and informal interactions.

Nursing schools have changed as they have adopted new learning paradigms, moving from transitional teaching models toward more innovative Social Constructivist models^(10)^. These changes not only affected teaching methods but also triggered identity transformations among nurse educators, extending beyond their teaching approaches and interpersonal relationships^(17)^. Changes in teaching models within nursing and nurse education have also reshaped how nurses perceive the role of nurse educators in shaping their professional identity^(11)^. In this study, nurses sometimes identify nurse educators as leaders and at other times distance themselves from them, depending on the educational model adopted.

Identification with nurse educators appears weakened by the social representations nurses hold of the nurse educator profession, with differentiation centered on two assumptions: nurse educators’ lack of familiarity with and distance from care practice contexts, and the fact that nursing and nurse education are professions with different areas of practice. Difficulty integrating theory and practice is considered a universal challenge in academic and healthcare organizations, which require curricula and educational models that effectively bridge this gap^(1)^. To narrow or eliminate the theory-practice gap, greater emphasis should be placed on integrated clinical practice^(5)^. In addition, closer collaboration and coordination between nurse educators and nurse tutors or supervisors should be promoted^(6)^.

In some accounts, the theory-practice divide clearly remained unresolved, revealing identities grounded in theory disconnected from practice, attributed to nurse educators, and identities grounded in practice-informed knowledge, claimed by nurses. Some nurses even stated that they themselves were the product of this divide between theory and practice in nursing. Some of these explanations, however, stemmed from their perception that nurse educators relied on an application-oriented, traditional approach to teaching, which had led them, as students, to believe that this same approach should be reflected in their own practice.

Within the category Perceptions of the Nurse Educator Profession, accounts of differentiation point to both positive and negative appraisals of this professional group, showing that nurses sometimes expressed negative differentiation and at other times positive differentiation toward nurse educators.

Some nurses expressed negative differentiation toward their own professional group while showing positive differentiation toward nurse educators. Within nursing’s view of teaching, a predominant theme emerged: nurses tended to converge with nurse educators, recognizing them as models and points of reference and also acknowledging their complementary roles. However, nurse educators who follow a traditional teaching and learning paradigm tend to foster a passive stance, positioning students as mere recipients of knowledge^(4)^. Within this paradigm, nurse educators are perceived as aligned with transmissive teaching^(4)^.

The perception of nurse educators as the exclusive holders of crucial knowledge has implications for professional identity, fostering more passive behaviors aligned with a paternalistic view^(18)^. Although this influence may initially take a traditional form, it also opens pathways and opportunities for genuine leadership in interactions with nurses, promoting a more autonomous and participatory professional identity^(5)^.

When nurse educators are understood as mentors, they play a significant role in reshaping relationships among all those involved in the educational process, including peers, nurses, and students^(19)^. This view underscores the need for collaborative efforts, innovative organizational structures, and new teaching strategies. It also gives students a more autonomous and proactive role, as they take responsibility for their own learning path and actively participate in the shared construction of knowledge^(20)^.

Traditional forms of teaching within the Traditional Model, and the relationships associated with them, were identified as sources of opposition to nurse educators. More innovative approaches, such as the Social Constructivist Model, by contrast, gave rise to positive identifications within this professional group. This trend aligns with the social constructivist paradigm, which underpins learning and enables nurse educators to exercise both formal and informal leadership roles^(8)^. This paradigm fosters critical-reflective capacities among nurses and facilitates a transformative process that contributes to reshaping their own professional identity in a more integrative, autonomous, and participatory direction^(21)^. In this way, it fosters the development of leadership competencies among nursing professionals.

In this study, when nurse educators are aligned with the Social Constructivist Model and foster more proactive forms of learning, they can exercise formal leadership by promoting critical reflective thinking, innovation, change, and autonomy in thought and practice, thereby helping develop future clinical leaders with leadership competencies. In the workplace, together with the ongoing pursuit of knowledge and experience, these competencies are essential to the development of the profession. For this reason, nurses perceived these educators as agents of transformation in care environments, professional development, and identity formation. Constructivist teaching values sociocultural influences on learning and supports knowledge construction based on prior experiences, rather than focusing exclusively on knowledge transmission, as in traditional formal education^(5)^.

Participants mentioned innovative approaches that connect learning contexts with practical care settings in “reference” communities as ways that not only facilitate shared professional and disciplinary development and the integration of theory and practice in nursing, but also create opportunities for nurse educators to exercise informal leadership^(19)^. This engagement promotes mutual knowledge exchange between nurse educators and nurses, fostering collaborative growth. As a result, nurses are better able to integrate clinical expertise with leadership in care, enabling them to exercise informal leadership among their peers^(19)^ and to act as innovative professionals and agents of change^(22)^.

This study recognizes and affirms the presence of formal and informal leaders among nurse educators, in line with previous findings^(2,8)^. These leaders, identified within the Social Constructivist Model, act as catalysts for change by promoting the development of leadership competencies in nursing education and by establishing partnerships with specialist clinical nurses. The feasibility and relevance of these partnerships are reinforced by the perceptions expressed in this study’s findings.

Nurse educators demonstrated various forms of leadership throughout nurses’ education, as reflected in expressions of critical-reflective thinking, in the promotion of learning environments grounded in closeness, trust, and autonomy in students’ thinking and actions, and in their ability to offer a broader perspective on care and its purposes-leadership in caring^(21)^.

Nurses recognized leadership among some nurse educators and expressed a desire for greater integration between these actors and “reference” communities, within collaborative and participatory climates regarded as essential to transformative interactions^(23)^. This desire for closer collaboration between nurse educators and nurses is also shared by nurse educators themselves^(24)^, alongside the aspiration to create “innovation and development centers” involving these stakeholders and bringing nurses and practice settings into research projects. “Learning communities” are crucial to identity reconfigurations and professional development because they enable shared reflection among all those involved, including the person receiving care, peers, members of the multiprofessional team, and students^(23)^, as this study further underscores.

From the perspective of a practice-based learning and “reference” community envisioned by nurses and nurse educators^(6,7,23,24)^, and recognizing the importance of formal leadership exercised by nurse educators and its potential to influence decision-making in healthcare settings and care practices^(2)^, professional knowledge is renewed and further developed. As a result, nurses’ identities are shaped through the sharing of new scientific evidence, the conduct of research, and collaborative education between academics and nurses, all of which are recognized as components of the Social Constructivist Model. Within this partnership, leadership dispositions are cultivated among professionals from diverse learning and clinical practice contexts^(25)^.

### Study limitations

This study has limitations related to participant selection, as the use of purposive sampling may affect the results and limit the generalizability of the findings. Future research should use more diverse sampling strategies to ensure broader representation. Even so, one of the study’s strengths is its in-depth analysis of how nurses in specialized care and primary care perceive academic nursing and educational models, offering valuable insights into the educator-student relationship and its impact on practice.

### Contributions to the field of nursing, health, or public policy

This study makes important contributions to nursing education by highlighting the persistent gap between theoretical knowledge and its application in practice. Nurses perceived a mismatch between the work of nurse educators, who were often seen as detached from clinical realities, and the practical demands of professional practice. This disconnect underscores the need for a more integrated approach to nursing education, one closely connected to practice-based experience. The findings suggest that stronger collaboration between nurse educators and nurses may help bridge this gap and foster a more cohesive learning experience. The study also highlights the need for nurse educators to engage more directly with clinical contexts and healthcare practice settings. Greater involvement of this kind would enrich teaching with real-world knowledge and more effectively support the professional identity formation and development of nursing students and, ultimately, nurses.

Examining education through the Traditional Model and the Social Constructivist Model provides a broad view of changes in nursing education. The study also highlights the need to further develop the Social Constructivist Model in curricula, nurse educator preparation, and pedagogical practice. This objective will require partnerships that promote leadership within communities of practice and encourage collaborations leading to innovative approaches, quality care initiatives within the nursing profession, and excellence in nurse education.

## FINAL CONSIDERATIONS

Although the interviewed nurses identified influences from nurse educators associated with both traditional and social constructivist models, the Social Constructivist Model proved more conducive to the exercise of leadership by nurse educators and to leadership development among nurses, as it enables the passive student/nurse, positioned merely as a recipient, to become a critical, reflective, and transformative student/nurse. This transition enables nurses to engage actively in their own professional development and helps strengthen a culture of leadership within the nursing community. Nurse education has been evolving to support the development of knowledge and expertise. The complexity of nurse education requires structural changes across the curriculum, nurse educator preparation, the educational model itself, and its various components. In this context, supervised clinical practice stands out because it expands learning beyond the classroom by incorporating clinical practice settings and promoting the integration of theory and practice.

## Data Availability

The research data are available only upon request.
