# Supplementary material for: Portuguese nurses’ perceptions of how educational models influence professional identity and practice
Source: Rev Bras Enferm. 2026 Sep 18;79:e20250262. doi: 10.1590/0034-7167-2025-0262 (PMC13588628; doi:10.1590/0034-7167-2025-0262)
Supplement: Translation

# Translation: Percepção de enfermeiros portugueses sobre influência dos modelos de formação na identidade e prática profissional

**DOI:** 10.1590/0034-7167-2025-0262pt

**Authors:** Sandra Rita Pereira Fernandes, Márcia Maria Carneiro Oliveira, Maria de Fátima Carneiro Pereira, Maria João Cardoso Teixeira, Olga Maria Pimenta Lopes Ribeiro, Tânia Marisa Pinto Rodrigues, Cânia Patrícia Torres, Beatriz Rodrigues Araújo

**Affiliations:** I Universidade do Porto. Porto, Portugal; II Universidade Federal da Bahia. Salvador, Bahia, Brasil; III Royal National Orthopaedics Hospital NHS. London, Reino Unido; IV Universidade Católica Portuguesa. Porto, Portugal

**Keywords:** Liderança, Enfermagem, Docentes, Ensino, Identidade Profissional.

## Abstract

**Objetivos:**

compreender como os modelos de formação docente tradicional e socioconstrutivista influenciam a construção da identidade profissional e a prática clínica de enfermeiros portugueses.

**Métodos:**

estudo descritivo, qualitativo, orientado pela teoria ego-ecológica de Zavalloni e Louis Guérin. Os dados derivaram da exploração das respostas ao Inventário de Identidade Psicossocial nas unidades representacionais de identificação e diferenciação com o grupo de docentes de Enfermagem, complementada por entrevistas conduzidas segundo as técnicas recomendadas.

**Resultados:**

os enfermeiros identificaram-se com docentes do Modelo Socioconstrutivista e se diferenciaram ou opuseram aos do Modelo Tradicional. Os achados permitiram reconhecer as lideranças exercidas e os obstáculos presentes nos grupos de diferenciação e identificação.

**Considerações Finais:**

no Modelo Socioconstrutivista, a influência docente sobre a identidade e prática clínica dos enfermeiros mostra-se mais efetiva. O estímulo a um papel crítico e transformador favorece o desenvolvimento profissional e fortalece uma cultura de liderança voltada à qualidade do cuidado e à inovação.

## INTRODUÇÃO

No contexto atual da saúde, em que a complexidade e exigência de inovação e desenvolvimento são necessários para prestação de cuidados de enfermagem de qualidade, os docentes de Enfermagem são essenciais para o processo de crescimento do estudante no seu espírito crítico, na procura de saber e na articulação entre teoria e prática. A dificuldade dessa articulação dentro da formação em enfermagem ainda é uma desafio constante, que pode ser superado com esforços colaborativos entre docentes, enfermeiros e estudantes^(1)^. Os docentes podem desempenhar o papel de liderança na relação com os estudantes e enfermeiros dos serviços, influenciando a prestação de cuidados centrados na pessoa e baseados em evidências, bem como fortalecendo parcerias com enfermeiros pesquisadores da prática clínica^(2)^. No exercício de uma liderança efetiva, esses docentes devem estar conscientes do seu papel de referência ou modelo. Eles o demonstram por meio de seu comportamento ético, da postura assertiva no ensino de responsabilidades profissionais e da forma de interagir com os pacientes, enfermeiros e demais membros da equipe multiprofissional^(2)^.

Considerando as transformações em curso no sistema de saúde em Portugal, torna-se essencial compreender até que ponto as referências de ensino dos docentes, ancoradas no Modelo Tradicional ou no Modelo Socioconstrutivista, podem contribuir para reforçar a conformidade institucional à mercantilização da saúde ou para promover a autonomia profissional e o cuidado humanizado^(3)^.

O Modelo Tradicional de ensino-aprendizagem é centrado no docente, que é a autoridade transmissora do conhecimento, de modo que o papel do estudante torna-se meramente passivo. No Modelo Socioconstrutivista, o estudante, o docente e o ambiente social estão envolvidos no processo de desenvolvimento do conhecimento^(4)^ e no ensino de enfermagem. Esse modelo enfatiza a valorização das expectativas, conhecimentos e experiências dos estudantes por meio de métodos de aprendizagem proativos, além de posicionar o docente como facilitador desse método^(5)^.

A colaboração entre docentes e enfermeiros durante as práticas de ensino clínico favorece um ambiente de partilha de conhecimentos e experiências, promovendo mudanças. As “comunidades práticas de aprendizagem” apresentam inúmeras vantagens associadas, como a aprendizagem em contexto, o envolvimento, a colaboração, a confiança e a integração entre docentes, estudantes e enfermeiros dos serviços^(6-8)^.

No ensino de enfermagem, as parcerias entre docente, enfermeiro supervisor e tutor, interligam saberes que, partilhados, são fundamentais à formação de futuros enfermeiros^(8)^. A prática do ensino clínico é importante para a socialização e construção da identidade profissional dos estudantes de Enfermagem, processo no qual os enfermeiros dos serviços desempenham papel fundamental, constituindo-se também como referências profissionais para os futuros enfermeiros em um ambiente colaborativo de aprendizagem^(7)^. Os docentes que lecionam segundo o Modelo Socioconstrutivista, ao exercerem e ensinarem liderança, isto é, influenciarem enfermeiros e futuros enfermeiros, favorecem que esses profissionais assumam seu potencial de liderança, promovendo inovação, mudança e melhorias na prestação de cuidados, nos serviços e no profissionalismo^(9)^.

Apesar de haver estudos sobre a necessidade de transformação do ensino de Enfermagem no Modelo de Formação Socioconstrutivista^(6,10-12)^, não foram encontrados trabalhos que reconhecessem, na percepção dos enfermeiros, a influência dos modelos de formação utilizados pelos docentes de Enfermagem sobre a identidade profissional e práticas de enfermagem.

A teoria ego-ecológica de Zavaloni e Louis Guerrin^(13)^ permite compreender a construção e reconstrução da identidade profissional de forma dinâmica e situada, considerando as interações do indivíduo com os diversos contextos sociais e institucionais em que se insere. Essa perspectiva inclui processos cognitivos como seleção e memorização da informação, assim como fenômenos de influência social e de atribuições. Assim, permite-se a ligação do passado, presente e futuro, enquanto recodificação, avaliação e orientação no ambiente profissional dos enfermeiros - espaço das realizações e dos insucessos. Conteúdos subconscientes permanecem na periferia da consciência, mas influenciam as trajetórias identitárias e as suas práticas. Esse referencial mostra-se particularmente adequado para analisar as influências dos diferentes modelos de formação utilizados pelos docentes sobre a construção da identidade e prática profissionais de enfermeiros portugueses, integrando de forma articulada as dimensões individual, relacional e ecológica.

Diante do exposto, coloca-se a seguinte questão de investigação: “Como os modelos de formação docente influenciam a construção da identidade profissional e a prática clínica dos enfermeiros portugueses?”.

## Relevância do estudo

Este artigo é produto da Tese de Doutoramento^(14)^ depositada no repositório institucional (http://hdl.handle.net/10400.14/36698).

## OBJETIVOS

Compreender de que modo os modelos de formação docente tradicional e socioconstrutivista influenciam a construção da identidade profissional e a prática clínica de enfermeiros portugueses.

## MÉTODOS

### Aspetos éticos

Esta pesquisa está de acordo com os princípios éticos para a Investigação Médica em Seres Humanos da Declaração de Helsinque, foi aprovada pela Comissão de Ética da Universidade Católica Portuguesa e seguiu todos os procedimentos éticos associados. O Consentimento Livre e Esclarecido foi obtido de todos os indivíduos envolvidos no estudo após a leitura da informação da pesquisa e esclarecimento de dúvidas. A participação ocorreu de forma voluntária, mediante concordância expressa e assinatura do termo de consentimento.

### Referencial teórico-metodológico

O estudo baseou-se na teoria ego-ecológica, que concebe o indivíduo como inserido em um meio social marcado por pertenças e interações com grupos sociais, no qual a memória emocional interna é influenciada por processos de identificação (*Self*) ou diferenciação (*Alter*) em relação aos outros do meio envolvente. Dessa perspectiva, enfatiza-se o impacto da identificação com o outro no ambiente imediato^(13)^. Ao adotar esse enquadramento, o estudo permitiu explorar como os docentes contribuem para a formação da identidade dos enfermeiros, influenciando suas experiências emocionais, crenças e autopercepções. Assim, possibilitou compreender a dinâmica emocional no ambiente de aprendizagem acadêmica e seu impacto no desenvolvimento da identidade.

### Tipo de estudo

A pesquisa adotou uma abordagem qualitativa, com base na teoria ego-ecológica de Zavalloni e Louis-Guérin^(13)^. O estudo seguiu as diretrizes da Rede EQUATOR e utilizou o *checklist* COREQ.

### Amostra

Utilizou-se amostragem intencional, com técnica de bola de neve^(15)^ para a coleta de dados aprofundados. Participaram 19 enfermeiros tutores e/ou supervisores que atendiam aos seguintes critérios de inclusão: ter dez ou mais anos de serviço e exercer funções em cuidados especializados ou na atenção primária em Portugal. A técnica de bola de neve foi iniciada com dois contatos iniciais, que indicaram outros profissionais de sua rede, permitindo expandir progressivamente a amostra e incluir enfermeiros de diferentes instituições, com diversidade de experiências. A entrevistadora responsável por todas as entrevistas tinha experiência em estudos qualitativos desde 2004 e treinamento na metodologia ego-ecológica desde 2014. Era também docente com doutorado em Enfermagem e mantinha relação profissional com os dois primeiros entrevistados, colegas enfermeiros da instituição onde atuou como enfermeira.

### Instrumento de coleta de dados

Inicialmente, os participantes foram convidados por e-mail, ocasião em que receberam informações sobre o estudo. Após o consentimento, foram agendados os locais e as datas dos encontros segundo as preferências dos participantes. Em cada encontro, estiveram presentes apenas o investigador principal/entrevistador e o entrevistado. Os locais variaram entre domicílios, automóveis e salas dos serviços.

No dia da recolha de dados, solicitou-se o preenchimento do Inventário de Identidade Psicossocial (IIP) adaptado^(16)^, utilizado como estímulo para as respostas e, em seguida, procedeu se à sua exploração mediante entrevista com duração entre 60 e 240 minutos. Por meio do IIP, solicitou-se a cada entrevistado que se identificasse ou se diferenciasse em relação aos grupos “Nós...” e “Eles...”. Para este estudo, o “Nós” correspondeu aos enfermeiros; e o “Eles”, ao grupo dos docentes de Enfermagem com os quais interagiram ou interagem. Dessa caracterização, emergiram as unidades representacionais, ou dados de primeiro grau, que identificam ou diferenciam o *Self* (enfermeiro) do *Alter* (docente de Enfermagem).

Essas associações visam suscitar sequências de conteúdo entre o pessoal e o coletivo, usadas para evocar novos conteúdos^(13)^. Posteriomente, seguiu-se a técnica indicada pelos autores do inventário, e os dados de primeiro grau serviram de estímulo para a entrevista, gerando dados de segundo e terceiro grau por meio da introspeção focalizada. Assim, foram reconhecidas as relações sociais com o Outro/*Alter*, selecionado pelos enfermeiros como o docente de Enfermagem, e suas influências emocionais na construção do *Self* profissional enfermeiro.

No Meio Interior Operatório do sujeito, armazena-se a memória de longo prazo, que emerge em condições mínimas de estimulação. O acesso a ela ocorre mediante a significação desses objetos de representação de *Si* e do *Outro*, que se consegue por meio das diferentes significações de pertença e diferenciação de valência positiva, negativa ou neutra, subgrupos, de aplicação a *Si* e de importância dessas representações, com uso do Método de Contextualização Representacional (MCR)^(13)^. Assim, a entrevista foi realizada segundo o MCR e a Análise de Redes Associativa. Utilizou-se o material produzido pelo sujeito, com elucidação do sentido para os dados apresentados e suas categorizações e representações, conseguindo fazer coincidir a história e a experiência pessoal com a coletiva mediante a compreensão dos processos de articulação do vivido pessoal com o vivido social. Desse modo, fazem emergir da consciência as unidades representacionais, que correspondem a imagens, pessoas, situações, lembranças, que habitualmente estão fora da consciência do sujeito, mas que surgem com os estímulos^(13)^. O procedimento repetiu-se até à obtenção da saturação dos dados.

### Análise dos dados

A análise dos dados seguiu a metodologia ego-ecológica, e o seu processo envolveu a categorização por meio de uma análise comparativa das entrevistas sob a perspectiva do Modelo de Formação Tradicional justaposto ao Modelo de Formação Socioconstrutivista^(4)^. Essa abordagem visava elucidar as dinâmicas subjacentes e os temas predominantes relativos às metodologias de formação percebidas pelos participantes. As entrevistas foram gravadas em áudio, identificadas de ID1 a ID19, transcritas na íntegra e devolvidas aos participantes para comentários ou alterações no prazo de duas semanas. Na ausência de comentários, assumiu-se que o participante concordava com o conteúdo. Foi efetuada uma análise temática por três pesquisadores de acordo com a metodologia de Braun e Clarke^(15)^, utilizando o software NVivo; e foram sistematicamente desenvolvidas categorias, subcategorias e códigos até se atingir a saturação dos dados^(12)^. Para garantir o rigor e a integridade, três autores - especialistas em análise temática há mais de dez anos e envolvidos em vários projetos de investigação relacionados com o ensino de Enfermagem - revisaram coletivamente as categorias e subcategorias derivadas da codificação e análise de dados, assegurando uma abordagem reflexiva e a integridade ao longo de todo o processo. O quadro final foi composto de duas categorias: Visão da Profissão de Docente e Vantagens e Desvantagens. Esta última foi subdividida em Competências Relacionais, Competências Formativas e Competências Científicas. O conjunto das categorias, subcategorias e respectivas codificações foi discutido e validado pelo restante da equipe de pesquisa.

## RESULTADOS

Na análise dos dados, observou-se maior percentual nas unidades de significação pertencentes às categorizações do Modelo de Formação Tradicional do que do Modelo de Formação Socioconstrutivista.

Foram convidados 19 enfermeiros tutores e/ou supervisores experientes, com diferentes formações acadêmicas e modelos de ensino, todos os quais aceitaram participar, resultando numa amostra final de 19 participantes. Treze tinham formação superior pós-graduada, enquanto seis tinham apenas licenciatura em Enfermagem. Os enfermeiros tinham uma média de 17,8 anos de serviço e uma média de 40 anos de idade, sendo que 13 trabalhavam em hospitais e 6 em centros de saúde.

Com uso do IIP, emergiram dados de primeiro grau, que refletiram processos de identificação e diferenciação positiva, negativa e neutra com o grupo dos docentes de Enfermagem. Com a análise das entrevistas, exploraram-se as evocações e emergiram significações ligadas às relações com os docentes, destacando-se o seu modelo de ensino. Observou-se identificação com os docentes de matriz socioconstrutivista e diferenciação em relação aos docentes de matriz tradicional de formação. O uso da teoria ego-ecológica^(13)^ permitiu perceber como a interação com os docentes e seus modelos de ensino, enquanto “Outros significativos”, sustenta as transições identitárias dos enfermeiros.

Mediante a análise, elaborou-se um diagrama (Figura 1) para melhor esquematização dos posicionamentos e conteúdos referidos pelos participantes.

**Figura 1:** Árvore de codificação resultante da análise temática, Porto, Portugal

Com base nas respostas ao Inventário da Identidade Psicossocial, foi possível perceber que os enfermeiros situam-se em um continuum entre diferenciação e identificação em relação aos docentes de Enfermagem, revelando predominantemente ambiguidades identitárias diante desse grupo profissional, associadas a seus modelos de formação praticados por esses docentes: Tradicional ou Socioconstrutivista.

### Modelo de Formação Tradicional

A análise dos dados possibilitou reconhecer que a diferenciação dos enfermeiros entrevistados predominou na forma como percebiam a profissão de docente de Enfermagem (Visão da Profissão de Docente) e nas Competências Relacionais, Formativas e Científicas associadas ao Modelo de Formação Tradicional, conforme esquematizado na Figura 1.

### Visão da Profissão de Docente

A diferenciação mostrou-se mais evidente na categoria Visão da Profissão de Docente, na qual os docentes foram percebidos como desconhecedores dos contextos de trabalho dos enfermeiros e inseridos em áreas distintas daquelas em que os enfermeiros atuam.

Os enfermeiros entrevistados diferenciaram-se dos docentes, principalmente, por considerá-los distantes e desconhecedores dos seus contextos de trabalho:

[…] *na generalidade, obviamente, que há exceções; na generalidade, os docentes de Enfermagem deixaram de ser enfermeiros sob o ponto de vista da prestação de cuidados.* (ID6)

[…] *é paradigmático que o enfermeiro que passe a dedicar-se em exclusivo à escola passe do ministério da saúde para o ministério da educação, o que é representativo do que é ser docente em Enfermagem: é alguém que está numa escola a ensinar Enfermagem, mas que acaba por ter um desfasamento* [uma defasagem] *daquilo que é a prestação direta de cuidados.* (ID10)

A separação entre a teoria e a prática não se mostra resolvida, evidenciando-se identidades apoiadas nas “teorias teóricas” atribuídas aos docentes e identidades sustentadas nas “práticas teóricas” reivindicadas pelos enfermeiros:

[…] *porque o teórico é aquele que sabe um corpo de conhecimentos e depois não consegue aplicar na prática, não consegue ser versátil, não consegue aplicar à pessoa que tem à frente, em diferentes contextos. A teoria é importante para um conjunto de corpos científicos e claro que é importante para aquilo que eu faço; agora um teórico é aquele que não consegue sair da teoria e dar-lhe sentido na execução prática.* (ID11)

*Precisamos de mais docentes que sejam enfermeiros e de menos docentes teóricos de enfermagem, que passaram imediatamente para a teoria, e aí há uma certa desadequação, e ficam um bocadinho aquém!* (ID8)

Segundo a percepção desses enfermeiros, no que diz respeito ao distanciamento em relação à prestação direta de cuidados, não se verificam consensos acerca da vantagem ou desvantagem dessa distância para o atual ensino de Enfermagem. Ainda assim, predominam unidades de significação que apontam para a dificuldade que esse distanciamento impõe ao ensino e ao estudante:

*Acho que a teoria e a prática deviam ser juntas, mas, neste momento, como os docentes só estão nas escolas a dar aulas, vejo que funciona melhor separadas*. (ID13)

*Atualmente, algumas escolas já estão a mudar de metodologias, já articulam o docente da escola com o orientador do serviço. Existe uma proximidade maior com o orientador do serviço e da prática; a maior parte dos estágios são orientados pelo docente da escola e por um supervisor ou tutor da prática.* (ID10)

Os enfermeiros chegam a constatar que eles próprios são resultado da separação entre a enfermagem teórica e a enfermagem prática, pois tinham a percepção de que os docentes solicitavam a mera aplicação da teoria à prática e não a articulação entre esses componentes do conhecimento, o que lhes provocou dificuldades, enquanto profissionais, no desenvolvimento de cuidados:

[…] *dá a sensação de que, quando tu estás com os docentes na escola, é tudo muito linear, que vais seguir aqueles passos todos direitinhos, daquele raciocínio, daquela linha de pensamento que “Eles” estão a tratar, mas depois, na realidade, tu chegas, e vai depender muito da pessoa que tens à frente, das tuas condições de trabalho, da liderança dos gestores do serviço.* (ID14)

*Há uma separação muito grande entre a teoria que ficou na escola e a prática, que depois deixaram de a perceber; e acho que “Nós” depois somos o fruto disto tudo, porque depois somos avaliados por alguém que não nos conhece, da parte do ensino, e que houve uma separação.* (ID18)

Na categoria Visão da Profissão de Docente, alguns entrevistados consideraram que as profissões de enfermeiro e de docente de Enfermagem são distintas, sobretudo por causa das diferentes áreas de atuação:

[…] *são dois grupos diferentes, porque trabalham em áreas diferentes, com sentidos diferentes. Um trabalha com o doente, a família como foco de cuidados; o outro tem como foco o estudante - difere no cliente!* (ID1)

*Não os considero completamente profissionais de saúde porque estão fora do contexto de prestação de cuidados. “Eles” ensinam a prestação, mas não a praticam. “Nós” somos enfermeiros que prestamos cuidados na nossa prática, e “Eles” são enfermeiros que não prestam esses cuidados, mas os ensinam.* (ID5)

### Déficits nas Competências Relacionais

As competências também constituíram uma forma de diferenciação entre os enfermeiros e os docentes de Enfermagem, predominando o déficit nas Competências Relacionais, seguido pelo déficit nas Formativas e nas Científicas no constructo Modelo de Formação Tradicional.

A diferenciação entre enfermeiros e docentes é evidenciada pela dinâmica das relações interpessoais e pelos métodos de avaliação.

Os enfermeiros referem-se à relação docente-estudante como distante, hierarquizada e rígida:

*Enquanto postura, em termos da forma como se relacionavam conosco, da pouca proximidade, daquilo que esperam de “Nós”, não há flexibilidade em relação ao estudante - é aquilo e tem que ser aquilo!* (ID 2)

*…as pessoas que estão lá acabam por achar que estão numa elite diferente das outras pessoas, mas não quer dizer que sejam tão melhores do que as outras. Há um bocadinho daquela dualidade entre escolas, aqueles conflitos que por vezes se transmitem para os profissionais.* (ID9)

O caráter ameaçador da avaliação é apontado como uma das causas do problema do distanciamento na relação:

[…] *ser colocada à prova, pressionarem, parece que nos estavam a humilhar* […]. (ID3)

[…] *enquanto estudante, tinha alguns medos, porque estávamos a ser avaliadas*. (ID4)

### Déficits nas Competências Formativas

A percepção sobre o déficit nas competências formativas foi identificada nos docentes, percebidos como transmissores de conhecimentos e desmotivadores. Os enfermeiros desvalorizavam um ensino baseado apenas no método transmissivo:

[…] *pouco dinâmicos, são aqueles que vão para lá só debitar as coisas* [despejar os conteúdos]*, os dispositivos e está feito!* (ID19)

[…] *tive alguns que pensei: com este não aprendi! Com este é só debitar!* (ID7)

Os participantes desta pesquisa distanciavam-se de docentes cujos comportamentos desmotivavam o estudante pelas críticas e pelo modo de ensinar:

*As críticas não eram motivadoras, não eram para construir algo de melhor. Eram críticas pouco motivadoras, críticas para, às vezes, desmotivar e desistir*. (ID4)

[…] *são aqueles docentes de Enfermagem que dizem coisas monótonas, não despertam interesse, que só despejam informação e de forma monocórdica.* (ID8)

### Déficits nas Competências Científicas

O distanciamento e a oposição foram observados nos docentes que não atualizavam os seus conhecimentos nos diferentes saberes de Enfermagem nem a planificação formativa:

[…] *era como se tivessem cristalizado no tempo, aquilo que era o conhecimento em enfermagem, o que é completamente contrário àquilo que “Nós” sabemos que se passa nos cuidados de saúde.* (ID6)

[…] *apercebia-me que havia docentes de Enfermagem que davam uma disciplina em um ano e, no outro ano, davam outra, e os apontamentos não eram criados por Eles, mas sempre repetidos sem atualizações durante anos*. (ID18)

### Modelo de Formação Socioconstrutivista

Com a análise dos dados, reconheceu-se que os enfermeiros se identificavam com os docentes fundamentalmente pela forma como percebiam a profissão de docente de Enfermagem e pelas Competências Formativas, seguidas das Relacionais e Científicas (Figura 1), em consonância com o Modelo de Formação Socioconstrutivista.

### Visão da Profissão de Docente

Verificaram-se duas linhas de identificação, que, apesar de semelhantes, apresentam diferenças. Por um lado, os enfermeiros que enxergavam os docentes como modelos os endeusavam e não apresentavam muito raciocínio crítico nessa idealização; por outro lado, os enfermeiros que tomavam os docentes como referências apresentavam um maior raciocínio crítico e o faziam com algum distanciamento.

Os discursos que definiram os docentes de Enfermagem como modelos expressaram uma diferenciação positiva, entendida como uma influência considerada relevante na formação do estudante e em sua vida:

[…] *quando Eu não sei, existe um conjunto de pessoas às quais Eu poderei perguntar e estudar, que, a priori, são detentoras de determinados conhecimentos que vão suprir as minhas necessidades conceituais; “Eles”, em princípio, terão esse conhecimento, já terão estudado, já terão desenvolvido conhecimentos ou estão na posse de alguns conhecimentos que Eu ainda não tenho!!* (ID17)

[…] *o ser docente exige um saber, um ensino de patamar superior a um curso de base, portanto considero que são pessoas que têm uma inteligência um pouco superior para conseguirem depois terem conhecimentos e depois adaptá-los e transmiti-los aos estudantes para que eles consigam aprender.* (ID12)

Os enfermeiros que viam os docentes como referências concebiam essa caraterística com sentido crítico, como forma de desenvolvimento, principalmente na transição para o mundo do trabalho:

[…] *o docente X foi efetivamente o meu modelo enquanto enfermeiro, até Eu chegar à conclusão de que não podia ser! Porque era alguém que estava tão ligado à parte do ensino e, durante tanto tempo, que já não consegui rever nele aquilo que era o meu paradigma de enfermeiro, embora continue a ser uma pessoa que Eu considero de notadíssima competência.* (ID6)

[…] *orientar na busca do conhecimento, como guias orientadores, como se nos pudessem ajudar a marcar o caminho.* (ID15)

Os enfermeiros referiram que se identificavam com os docentes porque os consideravam complementares, “Eles” possuíam um conhecimento “teórico”; e os enfermeiros, um conhecimento “prático”, logo as duas profissões complementavam-se, evidenciando-se não uma identificação com esses profissionais, mas uma diferenciação positiva:

*Os docentes carregam o conhecimento; quando falo em “Mestres”, falo de conhecimento teórico; já os enfermeiros são mais práticos. Os docentes podem ter muita teoria e muitos de “Nós”, também, têm muita teoria e vivem muito na prática e completam-se*. (ID7)

[…] *porque no fundo os docentes refletem aquilo que somos. São Eles que estão a formar os enfermeiros e contribuir para que sejamos profissionais qualificados.* (ID3)

### Competências Formativas

Nas Competências Formativas, os entrevistados identificavam-se com docentes motivadores e exigentes:

[…] *despertar curiosidade pelo tema; motivou e vais procurar algo sobre a temática ou te obriga a ir procurar!* (ID19)

[…] *faz com que haja uma maior motivação por parte dos seus estudantes, os tais que os potenciam, que os motivam, que os levam a crescer, a desenvolver os seus conhecimentos. Se “Eles” são instigantes, então desenvolvem a motivação.* (ID8)

Apesar de considerarem que, como estudantes, a exigência foi um fator de diferenciação, atualmente, como enfermeiros, essas exigências permitiram-lhes desenvolver a vontade de aperfeiçoar e melhorar, tornando-se um fator de identificação com os docentes.

### Competências Relacionais

A identificação com os docentes de Enfermagem, nas competências relacionais, reflete-se na proximidade estabelecida na relação. Os enfermeiros participantes valorizam uma relação de proximidade com os docentes, marcada por um percurso de desenvolvimento e pelo fortalecimento do potencial do estudante:

*É conheceres o estudante, teres uma certa proximidade, não uma proximidade de intimidade, mas uma proximidade para conheceres o estudante, conheceres as suas fraquezas e os seus pontos fortes, para poderes depois explorá-lo; isto, tanto em termos de ensino clínico como em termos da docência, ou então “Nós”, enquanto estudantes, motivar-nos nesse percurso evolutivo.* (ID1)

[…] *é respeitar o espaço da outra pessoa, respeitar a opinião da outra pessoa. E o respeito também é fundamental na relação docente-aluno.* (ID5)

### Competências Científicas

Nas Competências Científicas, as identificações centraram-se na Visão da Profissão de Docente, principalmente como detentor de conhecimentos e, depois, na atualização de conhecimentos. A competência científica, classificada inicialmente de identificação, passou a configurar-se como diferenciação positiva. Os enfermeiros designaram os docentes de Enfermagem como detentores de um saber que eles não tinham:

*Atualmente são pessoas portadoras de um saber diferente que “Nós” não temos. Têm mais estudos; em termos de saber, têm outro tipo de competência que “Nós” não temos*. (ID16)

*É uma pessoa que detém conhecimentos científicos e vai dar a Nós mais conhecimentos, não é? Mais capacidades!* (ID1)

A constante atualização dos conhecimentos foi reconhecida pelos enfermeiros como uma competência dos docentes com a qual se identificavam:

*Têm que estar em constante investigação, em constante produção de conhecimento, em constantes atualizações; têm que ter um trabalho muito proativo.* (ID9)

*Estão sempre em permanente atualização; estudam a mais recente evidência para ensinar.* (ID3)

## DISCUSSÃO

Esta pesquisa insere-se no paradigma do Construtivismo Social. Tem como referência a Teoria das Representações Sociais e, mais especificamente, a Teoria da Identidade Psicossocial de Zavalloni e Louis-Guérin, na qual as representações sociais são tidas como dinâmicas e em constante reformulação, influenciando os comportamentos^(13)^. Desse modo, na interação entre o “*Nós*” e o “*Eles*”, centra-se o conhecimento dos grupos de pertença e não pertença, isto é, as identificações e oposições/diferenciações, tal como as significações do sujeito. Além disso, o reconhecimento do *Outro* significativo torna-se importante para entender a identidade, as práticas e o desenvolvimento profissional.

Na vertente psicossocial de Zavalloni e Louis-Guérin^(13)^, está implicada uma abordagem contextual, integrativa de diversos fenômenos que interferem nas (re)configurações identitárias, desde a formação até o trabalho, integrando as mudanças nos contextos sociais, políticos, econômicos, e, especificamente neste estudo, os modelos de formação vigentes.

A formação, o trabalho e a identidade encontram-se interligados, desempenhando um papel na socialização e na qualificação para a profissão de enfermeiro durante a formação, o que permite a transição para o mundo do trabalho. Todavia, as influências da formação não se esgotam nesse período; essa influência formativa prolonga-se ao longo do desenvolvimento profissional, tanto em interações formais quanto informais.

As Escolas Superiores de Enfermagem modificaram-se ao adotarem novos paradigmas de aprendizagem, desde modelos transitórios de ensino até modelos mais inovadores socioconstrutivistas^(10)^. Essas mudanças não só tiveram impacto nos métodos de ensino, mas também desencadearam transformações identitárias nos docentes de Enfermagem, transcendendo suas abordagens de ensino e relações interpessoais^(17)^. As transformações nos modelos de ensino dentro da profissão de enfermagem e da docência em Enfermagem catalisaram mudanças na forma como os enfermeiros percebem o papel dos docentes na formação de sua identidade profissional^(11)^. Os enfermeiros deste estudo ora os identificam como líderes ora distanciam-se desses agentes educativos, conforme o modelo de formação adotado.

A identificação com os docentes de Enfermagem mostra-se fragilizada pelas representações sociais que os enfermeiros têm acerca da profissão de docente de Enfermagem, centrando as suas diferenciações em dois pressupostos: o desconhecimento/distanciamento dos docentes em relação aos contextos da prática assistencial e o fato de serem profissões com campos distintos de atuação. A dificuldade de articulação entre a teoria e a prática é considerada um desafio universal nas organizações acadêmicas e de saúde, as quais exigem currículos e modelos de formação que integrem eficazmente essa interligação^(1)^. Para atenuar ou erradicar o fosso entre teoria e prática, deve-se dar ênfase à prática clínica integrada^(5)^. Além disso, devem ser desenvolvidos esforços para promover uma colaboração e coordenação mais estreitas entre os docentes e os enfermeiros tutores ou supervisores^(6)^.

Em alguns discursos, torna-se evidente que o problema da separação entre a teoria e a prática não está resolvido, evidenciando-se identidades apoiadas nas “teorias teóricas” atribuídas aos docentes e identidades sustentadas nas “práticas teóricas” reivindicadas pelos enfermeiros. Alguns enfermeiros referem que eles próprios são o resultado do distanciamento entre a Enfermagem Teórica e a Enfermagem Prática. Contudo, algumas dessas justificativas decorrem do fato de perceberem os docentes como aqueles que ministram um ensino aplicacionista/tradicional, o que os induziu a pensar, quando eram estudantes, que essa abordagem deveria refletir-se em sua prática.

Na categoria “Visão da Profissão de Docente” de Enfermagem, os discursos de diferenciação tanto apontam para uma valorização quanto para uma desvalorização desse grupo profissional, constatando-se, por parte dos enfermeiros, ora uma diferenciação negativa, ora uma diferenciação positiva em relação aos docentes.

Alguns enfermeiros expressam uma diferenciação negativa em relação ao seu próprio grupo profissional, enquanto demonstram uma diferenciação positiva em relação aos docentes. No seio da perspectiva da profissão de enfermagem sobre o ensino, emerge um tema predominante em que os enfermeiros tendem a convergir para os docentes, reconhecendo-os como modelos e referências e reconhecendo também os seus papéis complementares. No entanto, os docentes que seguem um paradigma tradicional de ensino-aprendizagem tendem a incutir uma postura passiva, posicionando os estudantes como meros receptores de conhecimento^(4)^. Nesse paradigma, os docentes são percebidos como adeptos de um modelo de ensino transmissivo^(4)^.

A percepção dos docentes como detentores exclusivos de conhecimentos cruciais tem implicações na sua identidade profissional, promovendo comportamentos passivos mais próximos de uma visão paternalista^(18)^. Apesar de se manifestar inicialmente de forma tradicional, a influência dos docentes abre caminhos e oportunidades para o exercício de uma liderança genuína nas interações com os enfermeiros, promovendo uma identidade profissional mais autônoma e participativa^(5)^.

Na medida em que os docentes são definidos como mentores, desempenham um papel significativo na reformulação das relações entre todos os participantes no processo de formação, incluindo colegas, enfermeiros e estudantes^(19)^. Essa visão sublinha a necessidade de esforços colaborativos, de estruturas organizacionais inovadoras e de novas estratégias de ensino. Atribui também um papel mais autônomo e proativo aos estudantes, que assumem a responsabilidade pelo seu percurso de aprendizagem e participam ativamente na construção partilhada do conhecimento^(20)^.

As formas tradicionais de ensino do Modelo de Formação Tradicional e as relações a elas associadas foram identificadas como fontes de oposição aos docentes. Já abordagens mais inovadoras, como o Modelo de Formação Socioconstrutivista, suscitaram identificações positivas dentro desse grupo profissional. Essa tendência está alinhada com o paradigma socioconstrutivista, que serve de base à aprendizagem e capacita os docentes para exercerem papéis de liderança formais e informais^(8)^. Tal paradigma promove capacidades críticas e reflexivas entre os enfermeiros e facilita um processo transformador que contribui para a reformulação da sua própria identidade profissional, em uma direção mais integradora, autônoma e participativa^(21)^. Desse modo, fomenta-se o desenvolvimento de competências de liderança entre os profissionais de enfermagem.

Neste estudo, os docentes de Enfermagem, quando são enquadrados no Modelo Socioconstrutivista e desenvolvem formas de aprendizagens mais proativas, podem exercer lideranças formais, ao promoverem o pensamento crítico-reflexivo, a inovação, a mudança e a emancipação ou autonomia no pensamento e nas práticas, fomentando a existência de futuros líderes clínicos com competências de liderança. No contexto de trabalho, associadas à busca incessante do saber e às experiências, essas competências seriam essenciais para o desenvolvimento da profissão. Por isso, esses docentes foram percebidos pelos enfermeiros como agentes de transformação dos ambientes de cuidados, do desenvolvimento profissional e das identidades. O ensino construtivista valoriza as influências socioculturais no processo de aprendizagem e apoia a construção do conhecimento com base em experiências prévias, em oposição à preocupação exclusiva com a transmissão de conhecimentos, como na educação formal tradicional^(5)^.

As abordagens inovadoras que articulam os contextos de aprendizagem com os contextos práticos de cuidados em comunidades de “referência” foram mencionadas pelos participantes como formas que não só facilitam o desenvolvimento profissional e disciplinar conjunto e a articulação teórica e prática da enfermagem, mas também proporcionam oportunidades para os docentes exercerem liderança informal^(19)^. Esse envolvimento promove a troca mútua de conhecimento entre docentes e enfermeiros, favorecendo um crescimento colaborativo. Consequentemente, os enfermeiros são capacitados para integrar a expertise clínica com a liderança nos cuidados, permitindo-lhes exercer papéis de liderança informal entre os seus pares^(19)^ e atuar como profissionais inovadores e agentes de mudança^(22)^.

Este estudo reconhece e afirma a presença de líderes formais e informais entre os docentes de Enfermagem, em consonância com os resultados de investigações anteriores^(2,8)^. Esses líderes reconhecidos no Modelo de Formação Socioconstrutivista atuam como catalisadores da mudança, promovendo o desenvolvimento de competências de liderança no ensino da enfermagem e estabelecendo parcerias com enfermeiros clínicos especialistas. A viabilidade e a pertinência dessas parcerias são reforçadas pelas percepções expressas nos resultados deste estudo.

Os docentes demonstraram diversas formas de liderança ao longo da formação dos enfermeiros, exemplificadas por expressões de pensamento crítico e reflexivo, pela promoção de ambientes de aprendizagem baseados em relações de proximidade, confiança e autonomia no pensamento e nas ações dos estudantes, bem como pela capacidade de proporcionar uma perspectiva mais ampla sobre os cuidados e os seus objetivos - a liderança no cuidar^(21)^.

Os enfermeiros reconheceram que a liderança está presente em alguns docentes, manifestando o intuito de maior articulação entre esses atores e as comunidades de “referência”, em climas colaborativos e participativos, considerados essenciais para interações transformadoras^(23)^. O desejo de uma maior articulação entre docentes e enfermeiros também é sentido pelos docentes^(24)^, com a idealização da criação de “centros de inovação e desenvolvimento” constituídos por esses intervenientes e envolvendo os enfermeiros e as práticas nos projetos de pesquisa. As “comunidades de aprendizagem” são cruciais para reconfigurações identitárias e para o desenvolvimento profissional, permitindo a partilha e reflexão em conjunto com todos os atores intervenientes, incluindo a pessoa que é alvo de cuidados, pares, membros da equipe multiprofissional e estudantes^(23)^, como este estudo vem reforçar.

Na perspectiva de uma comunidade prática de aprendizagem e de “referência” idealizada e almejada pelos enfermeiros e pelos docentes^(6,7,23,24)^, e reconhecendo a importância da liderança formal por parte dos docentes e o seu potencial para influenciar a tomada de decisão nos contextos de saúde e nas práticas de cuidado^(2)^, os saberes profissionais são renovados e inovados. Consequentemente, as identidades dos enfermeiros são moldadas por meio da partilha de nova evidência científica, da realização de investigação e da formação colaborativa entre acadêmicos e enfermeiros, reconhecidas como componentes do Modelo de Formação Socioconstrutivista. Nessa parceria, cultivam-se atitudes de liderança entre profissionais oriundos de diversos contextos de aprendizagem e de prática clínica^(25)^.

### Limitações do estudo

O estudo apresenta limitações relacionadas à seleção dos participantes, devido ao uso de amostragem intencional, o que pode comprometer os resultados e a generalização dos achados. Pesquisas futuras deverão recorrer a métodos de amostragem mais diversificados, a fim de garantir uma representação mais abrangente. Ainda assim, entre os pontos fortes do estudo destaca-se a análise aprofundada das percepções de enfermeiros de cuidados especializados e de enfermeiros da atenção primária sobre a enfermagem acadêmica e modelos de formação, oferecendo contribuições valiosas para a compreensão da relação docente-estudante e do seu impacto na prática.

### Contribuições para a área da Enfermagem, saúde ou política pública

O estudo revela contribuições significativas para o ensino da enfermagem ao evidenciar a persistente distância entre o conhecimento teórico e a sua aplicação prática. Os enfermeiros perceberam um descompasso entre a atuação dos docentes, frequentemente vistos como afastados das realidades clínicas, e as exigências práticas do exercício profissional. Isso sublinha a necessidade de uma abordagem mais integrada no ensino da Enfermagem, estreitamente articulada com a experiência prática. Os resultados sugerem que o reforço da colaboração entre docentes e enfermeiros poderá ajudar a preencher essa lacuna, promovendo uma experiência de aprendizagem mais coesa. Além disso, a pesquisa evidencia a necessidade de os docentes se envolverem de forma mais direta com os contextos clínicos e com os cenários de prática dos serviços de saúde. Essa maior participação enriqueceria seu ensino com conhecimentos do mundo real e apoiaria de forma mais eficaz a construção da identidade profissional e o desenvolvimento dos estudantes de Enfermagem, consequentemente dos enfermeiros.

A análise da formação sob os Modelos Tradicionais e nos Modelos Socioconstrutivistas proporciona uma visão abrangente da evolução do ensino da enfermagem. O estudo destaca ainda a necessidade de desenvolver o Modelo de Formação Socioconstrutivista nos currículos, na formação docente e na prática pedagógica. Para isso, são necessárias parcerias que promovam a liderança nas comunidades de prática e incentivem colaborações que conduzam a abordagens inovadoras, a iniciativas de cuidados de qualidade no seio da profissão de enfermagem e à excelência na formação dos enfermeiros.

## CONSIDERAÇÕES FINAIS

Embora os enfermeiros entrevistados identifiquem influências de docentes de Enfermagem vinculados a modelos tradicionais ou socioconstrutivistas, o modelo socioconstrutivista mostrou-se mais favorável ao exercício da liderança pelos docentes e ao desenvolvimento da liderança nos enfermeiros, ao permitir que o estudante/enfermeiro passivo e receptor se torne um estudante/enfermeiro crítico, reflexivo e transformador. Essa transição capacita os enfermeiros a envolverem-se ativamente no próprio desenvolvimento profissional e contribui para fortalecer uma cultura de liderança dentro da comunidade de enfermagem. A formação dos enfermeiros vem se modificando, com vistas ao desenvolvimento de conhecimentos e saberes. A complexidade da formação dos enfermeiros exige mudanças estruturais no currículo, na formação dos docentes, no modelo de formação e em seus diversos componentes. Nesse contexto, destaca-se a prática clínica supervisionada, que amplia os espaços de formação para além da sala de aula, ao incorporar os cenários da prática clínica e promover a articulação entre teoria e prática.

## Data Availability

Os dados de pesquisa só estão disponíveis mediante solicitação.
